# A novel miniature transposon-like element discovered in the coding sequence of a gene that encodes for 5-formyltetrahydrofolate in wheat

**DOI:** 10.1186/s12870-019-2034-1

**Published:** 2019-11-01

**Authors:** Katherine Domb, Danielle Keidar-Friedman, Khalil Kashkush

**Affiliations:** 1 Present Address: Department of Molecular Biology and Ecology of Plants, Tel-Aviv University, Tel Aviv, Israel; 2 Department of Life Sciences, Ben-Gurion University, 84105 Beer-Sheva, Israel

**Keywords:** Miniature transposable elements, *Triticum*, *Aegilops*, 5-formyltetrahydrofolate, Genome evolution, Polyploidy, Wheat

## Abstract

**Background:**

Transposable elements (TEs) comprise over 80% of the wheat genome and usually possess unique features for specific super-families and families. However, the role of TEs in wheat evolution and reshaping the wheat genome remains largely unclear.

**Results:**

In this study, we discovered a miniature (307 bp in length) TE-like sequence in exon 6 of a gene that encodes for 5-formyltetrahydrofolate, in two accessions of wild emmer wheat (*T. turgidum* ssp. *dicoccoides*) and has interfered with the gene translation by creating a shorter reading frame as a result of a stop codon. The sequence that was termed *Mariam,* does not show any structural similarity to known TEs. It does not possess terminal inverted repeats (TIRs) that would allow us to assign this element to one of the TIR DNA super-families, and it does not possess characteristic features of SINE, such as a Pol-III promotor or a poly-A tail. *In-silico* analysis of five publicly available genome drafts of *Triticum* and *Aegilops* species revealed that *Mariam* element appears in a very low copy number (1–3 insertions) in diploid wheat species and ~ 12 insertions in tetraploid and hexaploidy wheat species. In addition, *Mariam* element was found to be unique to wheat, as it was not found in other plant genomes. The dynamic nature of *Mariam* in the wheat genome was assessed by site-specific PCR analysis and revealed that it retained activity in wild emmer populations in a population-specific manner.

**Conclusions:**

This study provides additional insight into the evolutionary impact of TEs in wheat.

## Background

Transposable elements (TEs) are DNA segments that have the ability to proliferate within their host, as such, they can make up large fraction of eukaryotic genomes [1]. The wheat genome harbor thousands of known TE families that occupy ~ 80% of the genome, while LTR retrotransposons are the most abundant [2–5]. TEs are divided into Class I elements (retrotransposons) and Class II elements (DNA transposons), which are further divided into super-families and families [6].

TEs can be stimulated by various biotic or abiotic stresses such as heat shock, wounding or bacterial infection [7–9], as well as genomic stresses such as hybridization and polyploidization [10]. Activity of TEs in the genome can alter its structure both genetically and epigenetically [11–14]. Insertions of TEs into coding regions (exons) can cause disruption of function and generation of mutant phenotypes [15], while insertions into introns can interfere with transcript editing or lead to intron retention [16–18]. Insertions of TEs in close proximity to genes can affect their expression, for example by interfering with promoter activity [15, 16]. One other known consequence of TEs amplification is expansion of the host genome size [14, 19]. All these effects have a key role in allelic and phenotypic diversity of plants [14].

Wheat (*Triticum-Aegilops* group) had originated ~ 4 million years ago following the divergence of three diploid species from a common ancestor. The tetraploid *T. turgidum* ssp. *dicoccoides* (wild emmer, AB genome) was generated by an allopolyploidization event that included hybridization of *Triticum urartu* (donor of A genome) and an unknown *Aegilops* species (of section *sitopsis*, donor of B genome). The primary domestication of wild emmer and the following evolution of hulled domesticated emmer wheat (*T. turgidum* ssp. *dicoccun*) induced the selection of free-threshing durum (*T. turgidum* ssp. *durum*, AB genome). The hexaploid *Triticum aestivum* (Bread wheat, ABD genome) was generated ~ 10,000 years ago by a second major polyploidization event that involved the hybridization between domesticated emmer wheat (*T. turgidum* ssp. *dicoccun*) and *Aegilops tauschii* (donor of D genome) [4, 20, 21]. Wild emmer is found in nature as a wild species and since its rediscovery by Aharon Aharonson in 1913 [22], this species has been extensively studied as a potential donor of beneficial traits to domesticated wheat [22–26]. Wild emmer, an annual, predominantly self-pollinating species, is distributed in a patchy manner throughout the Middle East in diverse environmental conditions that vary in average annual temperature, altitude, soil type, and other conditions. In Israel, there are over 20 populations (isolated or semi-isolated) of wild emmer that can be found in regions between Mt. Hermon in the north and Mt. Amasa (Judea desert) in the south [27, 28]. We recently showed that Transposable elements can proliferate in a population-specific manner in wild emmer wheat, thus creating allelic variation [29].

In this study, we present the discovery of a wheat-unique miniature TE-like sequence termed *Mariam* in two accessions of a marginal population of wild emmer wheat. The availability of 5 recently updated wheat genome drafts; *T. turgidum* ssp. *dicoccoides*, *T. turgidum* ssp. *durum*, *T. aestivum*, *Ae. Tauschii* and *T. urartu*, facilitated the computer-assisted analysis of *Mariam* content and dynamics in these wheat species. In addition, the impact of the novel *Mariam* insertion in a gene that encodes for 5-formyltetrahydrofolate was assessed, as well as the dynamics of *Mariam* in wild emmer wheat populations. To this end, the evolutionary impact of *Mariam* insertions is discussed.

## Results

### A novel TE-like DNA fragment discovered in the coding sequence of a gene that encodes for 5-formyltetrahydrofolate in Mt. Hermon population of wild emmer wheat

**Fig. 1:**
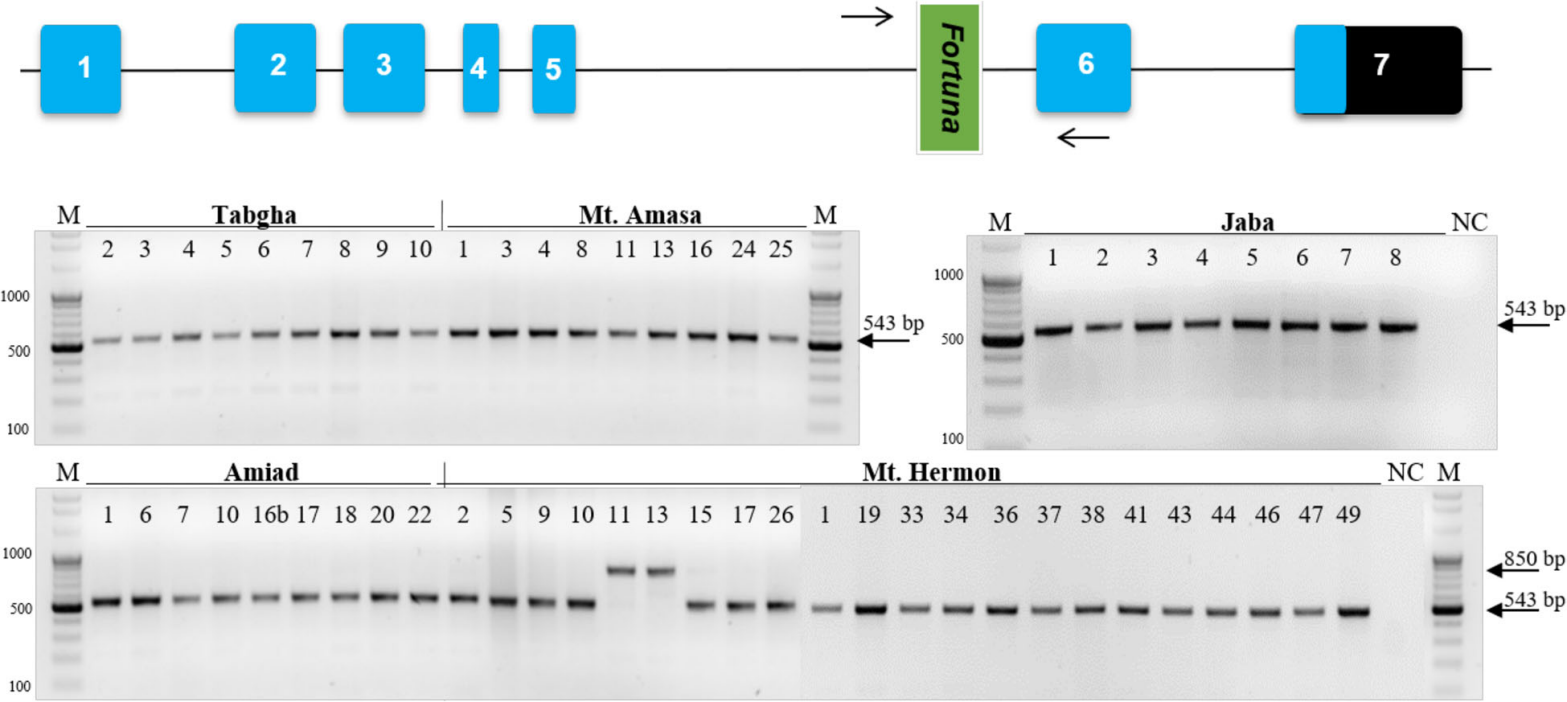
Top: Schematic structure of the TRIDC2AG023940 gene. The gene sequence consists of 7 exons (boxes) and a *Fortuna* TE between exon 5 and exon 6. Note that the coding sequence (CDS) of the gene ends in exon 7. Forward and reverse primers for ssPCR are indicated by arrows. Bottom: site-specific PCR analysis demonstrating *Fortuna* insertion in intron 5 of the TRIDC2AG023940 gene in all populations. The insertion is indicated by the lower band (543 bp) and a higher band (850 bp) in two accessions of the Mt. Hermon population (bottom left). M denotes a size marker (the numbers in the left are in bp), while NC denotes a negative control (water was used as template in PCR reaction). Primers (indicated in the top scheme) used for this ssPCR were: Forward = TCTTTGTGTATTCTCTAGCTCTGT and Reverse = ACTCCACCCTTTCTCTTTAGCA

As part of a study [29] that aimed to identify and characterize polymorphic insertions of miniature inverted-repeat transposable elements (MITEs) in five wild emmer wheat populations, we have discovered a short DNA insertion (307 bp in length) in exon 6 of a gene that encodes 5-formyltetrahydrofolate (TRIDC2AG023940, *EnsemblPlants*) in two accessions of Mt. Hermon population wild emmer populations (Fig. 1 top). Genome-specific primers were designed from intron 5 upstream to a MITE insertion, termed *Fortuna,* and from exon 6 of the gene (Fig. 1, Additional file 1: Table S1). The expected size of the full site was 543 bp. The site-specific PCR experiment showed that *Fortuna* element is present in all accessions of these five populations (Mt. Hermon, Amiad, Tabgha, Jaba and Mt. Amasa), yet in two accessions of the Mt. Hermon population, a higher band of 850 bp was amplified instead of the expected full or empty site (Fig. 1 bottom).

**Fig. 2:**
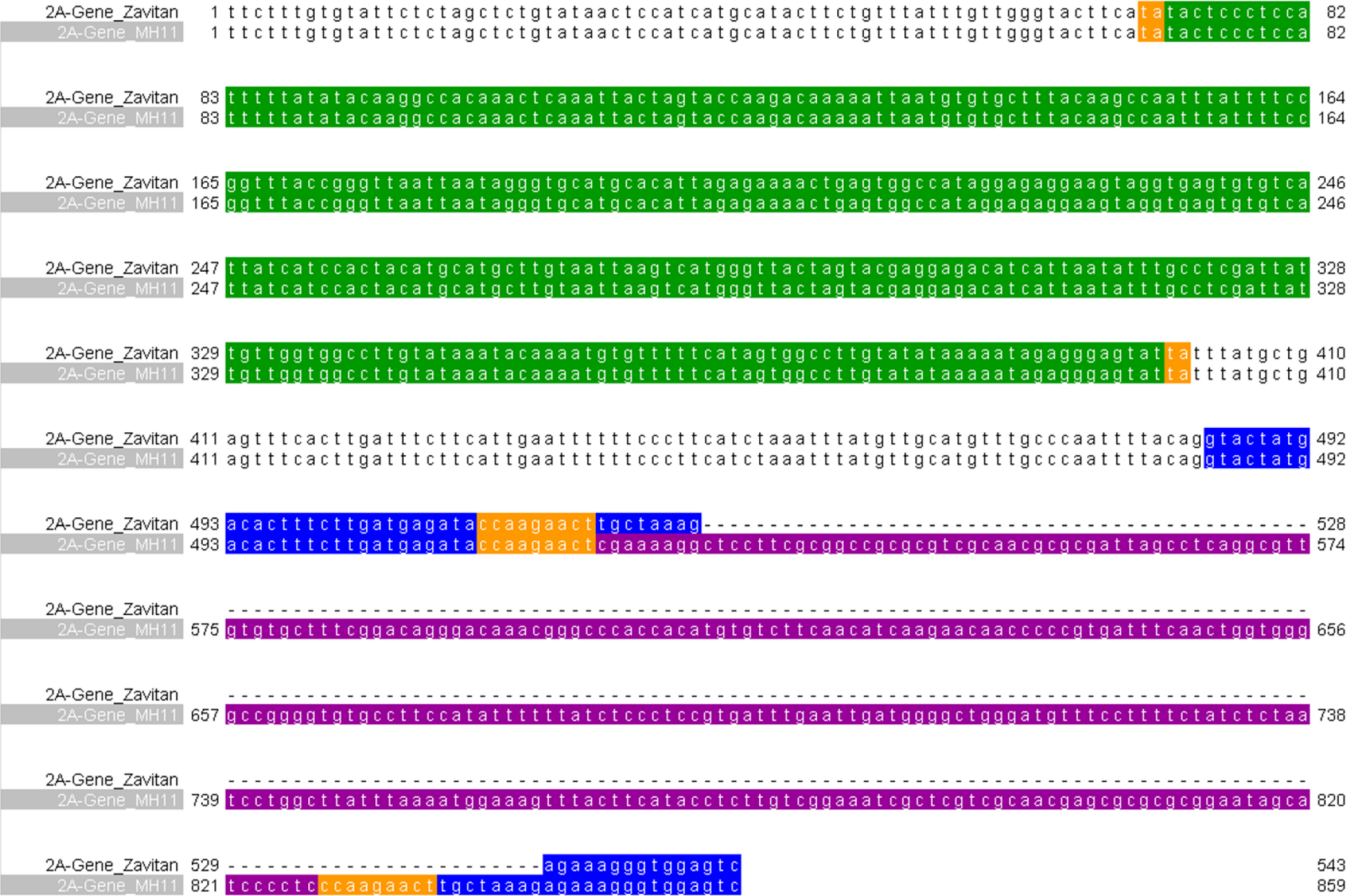
Pairwise sequence alignment of the TRIDC2AG023940 fragment (from accession Zavitan -top) and site-specific PCR product amplified in Mt. Hermon accession 11 (MH11-bottom). The colored sequences note: Green – *Fortuna* (MITE) insertion, purple – new insertion in exon 6, white – intron 5, blue – exon 6 upstream and downstream to the new insertion, orange – sequence duplications flanking insertions: 2 bp (TA) for *Fortuna*, and 9 bp (CCAAGAACT) for the new insertion

Sequence analysis of the 307 bp insertion (Fig. 2) revealed that this sequence did not hit any known sequence from the database including genomic and transcriptomic databases (https://plants.ensembl.org/Triticum_aestivum/Info/Annotation/ [30], http://plants.ensembl.org/Triticum_dicoccoides/Info/Annotation/ [4]), and repeat databases such as ITMI [31], the *Triticeae* repeats database (http://botserv2.uzh.ch/kelldata/trep-db/index.html), and GIRI database [32] (http://www.girinst.org/censor/index.php) that yielded no hits to annotated transposable elements. Interestingly, the 307 bp sequence was flanked by a 9 bp sequence (CCAAGAACT) at both ends resembling a target-site duplication (TSD) that can be generated as a result of transposable elements insertions (Fig. 2). The fact that the 9 bp site was found in only one copy in the gene lacking the new insertion indicates the 307 bp sequence was integrated in a TE-like manner, thus creating TSD. Furthermore, the 307 bp insertion does not possess terminal-inverted repeat sequences (TIRs) that would allow us to assign this sequence to one of the TIR DNA super-families. In addition, it does not possess characteristic features of SINEs such as a Pol-III promotor or a poly-A tail.

To test whether other copies of the TE-like insertion appear in *Triticum-Aegilops* genomes, MAK software was used to retrieve TE-like insertion sequences from the recently available genome drafts. We have found one insertion in *T. urartu* (AA genome), 3 insertions in *Ae. tauschii* (DD genome), 12 insertions (8 in subgenome A and 4 in subgenome B) in *T. turgidum* ssp. *dicoccoides* (wild emmer, AABB genome)*,* 6 insertions (4 in subgenome A and 2 in subgenome B) in *T. turgidum* ssp. *durum* (durum, AABB genome) and 11 insertions (3 in subgenome A, 4 in subgenome B and 4 in subgenome D) in *T. aestivum* (bread wheat, AABBDD genome). Sequences of all retrieved insertions and their molecular charactrization, including sub-genome and chromosmal locations can be found in Additional file 1: Table S1. Note that this insertion was found to be unique to *Triticum-Aegilops* group as it was not found in other plant genomes.

A sequence logo was created from the flanking sequences of the 33 full-length 307 bp insertions retrieved from the 5 genome drafts using WebLogo software [33] (http://weblogo.threeplusone.com/, Additional file 4: Figure S1). While 16 of the 33 insertions showed clear TSD, the remaining 17 insertions did not show notable TSD (Additional file 1: Table S1). The sequence logo demonstrated a certain sequence preference for specific nucleotides at positions 4 and 9, however the 9-bp duplicated sequences were not highly conserved. The lack of detectable TSDs for 17 insertions can be explained by mutations /rearrangements around the insertions that might have disrupted the formerly identical sequences flanking each insertion. Note that over 50% (9 out of 17) of the insertions lacking TSD are located in genome BB, which is known to be very dynamic [20, 21]. To this end, the fact that clear TSDs of conserved length were detected in ~ 50% of the insertions supports the hypothesis of a transposon-like behaviour, therefore we named this TIR-less, minature element “*Mariam”*. Note that multiple sequence alignment analysis revealed high sequence conservation (over 90%) of the 33 full-length *Mariam* elements retreived from the five genome drafts. The high sequence conservation of *Mariam* among diploid and polyploid species might strongly indicate a recent prolifiration of this element in wheat.

### Insertional polymorphism of *Mariam* in *Triticum-Aegilops* group and within wild emmer populations

As mentioned above, the existence of *Mariam* elements in *T. urartu* and *Ae. tauschii*, and in the three sub-genomes of bread wheat suggests this element was probably present in all diploid progenitors of the wild emmer and bread wheat and was amplified in the polyploid species. Of the 7 *Mariam* insertions found in A and B sub-genomes of *T. aestivum*, 6 were common (monomorphic insertions) to wild emmer. In addition, all 3 *Mariam* insertions found in *Ae. tauschii* were common to the D sub-genome of *T. aestivum*. Finally, the insertion found in *T. urartu* was common to wild emmer and to *T. aestivum*. The dynamics of *Mariam* elements in wild emmer wheat and in bread wheat accessions were assessed using site-specific PCR analysis on DNA isolated from 45 wild emmer accessions (collected from 5 different goegraphically isolated populations; Mt. Hermon, Amiad, Tabgha, Jaba and Mt. Amasa – 9 accessions from each population) and 8 bread wheat accessions (see Additional file 2: Table S2). Overall, primers were designed from flanking sequences of 10 *Mariam* insertions mapped to A and B sub-genomes of wild emmer and/or bread wheat (Additional file 3: Table S3).

**Fig. 3:**
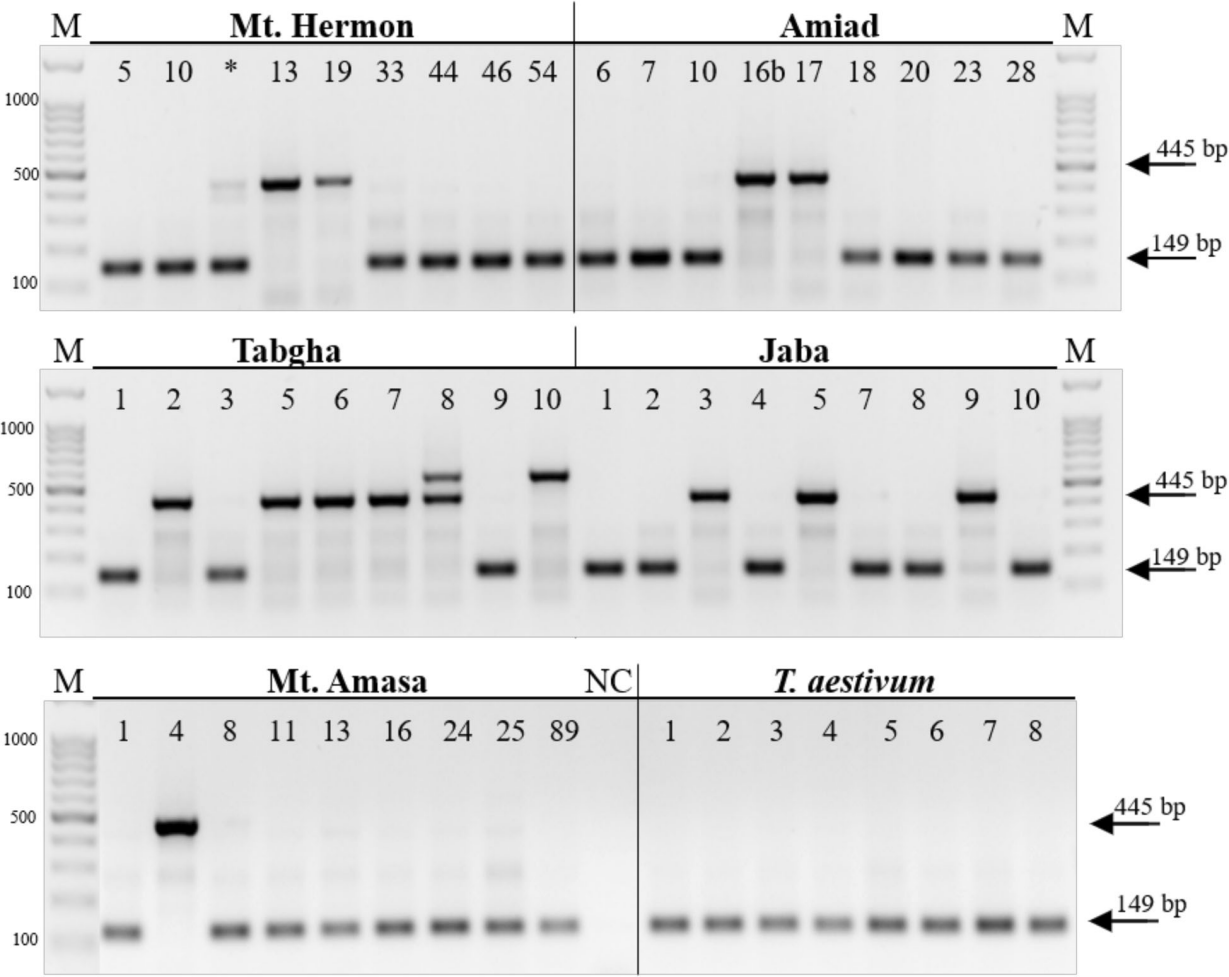
Site-specific PCR analyses of *Mariam* insertion A4–4 (see Additional file 1: Table S1). The PCR analysis was done using primers flanking the insertion in accessions collected from five populations of wild emmer wheat (Mt. Hermon, Amiad, Tabgha, Jaba, and Mt. Amasa) and in eight accessions of *T. aestivum* collected from different countries (1 – Georgia, 2 - Iran, 3 - USA, 4 – China, 5 - UK, 6 - Spain, 7 - Turkey, 8 – former Yugoslavia, see Additional file 2: Table S2). This insertion was polymorphic in all wild emmer wheat populations, while monomorphic (empty site) in all bread wheat accessions. Each accession presents either a 445 bp (full site) or a 149 bp band (empty site). M denotes a size marker (the numbers in the left are in bp), while NC denotes a negative control (water was used as template in PCR reaction)

The results of the PCR analysis demonstrated high insertional polymorphism levels of *Mariam* based on presence (full site) vs. absence (empty site) among wild emmer wheat accessions. Only one of the examined insertions, A5–6 (Table 1), was present in all accessions of wild emmer and bread wheats (monomorphic insertion), while 6 insertions (A4–4 (Fig. 3), A6–2, A7–5, B3–4, B7–4, B7–6 (Additional file 4: Figure S2) were polymorphic in wild emmer wheat accessions in a population-specific manner and were not detected in the bread wheat accessions (Table 1). One insertion (B1–4, Table 1) was absent from all wild emmer wheat accessions but was present in all bread wheat accessions. In two additional cases (A4–1 (Additional file 4: Figure S2f), B1–4 (Additional file 4: Figure S2g)), insertions were polymorphic in both wild emmer wheat accessions and in bread wheat accessions. A4–1 insertion exists in only one of eight bread wheat accessions, while B1–4 insertion exists in seven of eight accessions.

**Table 1:** Insertional polymorphism of *Mariam* in wild emmer wheat and in bread wheat accessions based on ssPCR analysis

Locus^a^	Presence (full site)/ absence (empty site) of *Mariam* in five wild emmer populations (9 accessions from each population) and in 8 bread wheat accessions^b^					
	Wild emmer populations (*T. turgidum* ssp. *dicoccoides*)					Bread wheat (*T. aestivum*)
	Mt. Hermon	Amiad	Tabgha	Jaba	Mt. Amasa	
A2-MH	1	0	0	0	0	0
A4–1	0	1	1	0	0	1
A4–4	1	1	1	1	1	0
A5–6	2	2	2	2	2	2
A6–2	0	1	1	0	0	0
A7–5	0	1	1	0	1	0
B1–2	0	0	0	0	0	2
B1–4	1	1	2	1	1	1
B3–4	0	1	1	0	0	0
B7–4	0	1	1	1	0	0
B7–6	0	1	1	0	0	0

^a^The chromosome where the insertion was found, and an additional identifier of each insertion site

^b^The presence/absence of a TE insertion in examined accessions of a given population (for *T. dicoccoides*) or a given species (*T. aestivum*): 0 – empty site in all accessions, 1 - full site in some accessions, 2 – full site in all accessions. See Figs. 1 and 3 and Additional file 4

**Fig. 4:**
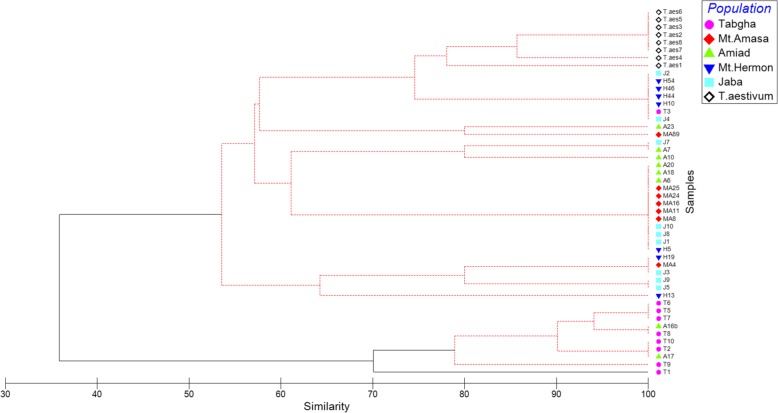
Phylogenetic tree generated by multi-dimensional scaling based on presence/absence of 11 *Mariam* insertions. The tree was built according to the PCR analysis of 40 accessions of wild emmer wheat populations from Mt. Hermon, Amiad, Tabgha, Jaba and Mt. Amasa and 8 accessions of bread wheat (indicated on the box). Black lines indicate significant separation, while red lines indicate insignificant separation. The level of similarity is indicated on the bottom

The insertional pattern of *Mariam* (according to the results of the ssPCR analysis) was used to build a phylogenetic tree generated by hierarchical agglomerative clustering (see Fig. 4). The phylogenetic tree showed significant (*p*-value< 0.05) separation between Tabgha population (except for T3 accession) and other populations, which correlates with our previous finding according to *Minos* (a MITE family) insertional pattern that was assessed Transposon Display assay [29]. All *T. aestivum* accessions were clustered together, as well as Mt. Amasa and Amiad that were generally clustered by their population, except for two of their accessions. Jaba and Hermon populations showed high variability among accessions within population. These findings might indicate the population-specific dynamics of *Mariam* in wild emmer wheat and in bread wheat species.

### *Mariam* insertions are associated with wheat genes

Sequence annotation of *Mariam* insertion sites revealed that 11 of the 33 *Mariam* insertions were located adjacent to other TE elements including class I and class II elements (Additional file 1: Table S1), while 12 insertions were located into or adjacent (up to 500 bp downstream or upstream) to genes, such as formyltetrahydrofolate cyclo-ligase, sucrose-phosphatase 3, soleucyl-tRNA synthetase, Serine/threonine-protein kinase, xyloglucanase and others. This is in addition to the previously described *Mariam* insertion (A2-MH) in exon 6 of the TRIDC2AG023940 gene encoding a predicted mitochondrial 5-formyltetrahydrofolate cyclo-ligase.

**Fig. 5:**
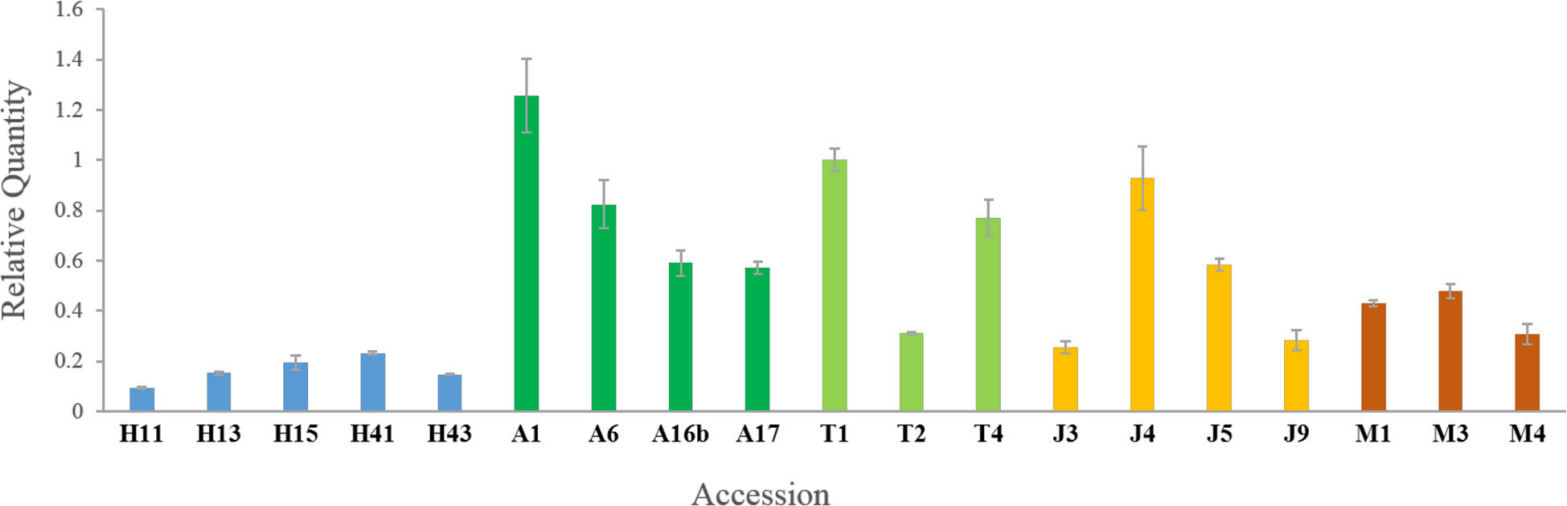
Relative Expression levels of TRIDC2AG023940 gene revealed by real-time RT-PCR, in wild emmer wheat accessions. Primers were designed to amplify the junction between exons 1 and 2 (see Methods). Wild emmer wheat accessions from five populations were analyzed (H – Mt. Hermon, A – Amiad, T – Tabgha, J – Jaba, M – Mt. Amasa). The results demonstrate the relative expression levels of the gene in the various accessions vs. the gene expression level in accession T1 (set as 1). For statistical analysis (see Methods), the average expression levels of the gene in each population was calculated and found no significant differences among Amiad, Tabgha, Jaba, and Mt. Amasa, while the expression level of the gene in Mt. Hermon was significantly lower than that of Amiad

To test whether *Mariam* insertion in the gene coding sequence has any impact on the gene activity, we designed primers for RT-qPCR analysis from TRIDC2AG023940 gene and/or its homolog from the B genome. The relative expression level of each accession was normalized to the relative expression level of an arbitrary chosen accession (T1) to enable comparison between samples. The relative expression levels of TRIDC2AG023940 and/or its homolog from the B sub-genome were found to vary within and between populations of wild emmer wheat (Fig. 5).

**Fig. 6:**
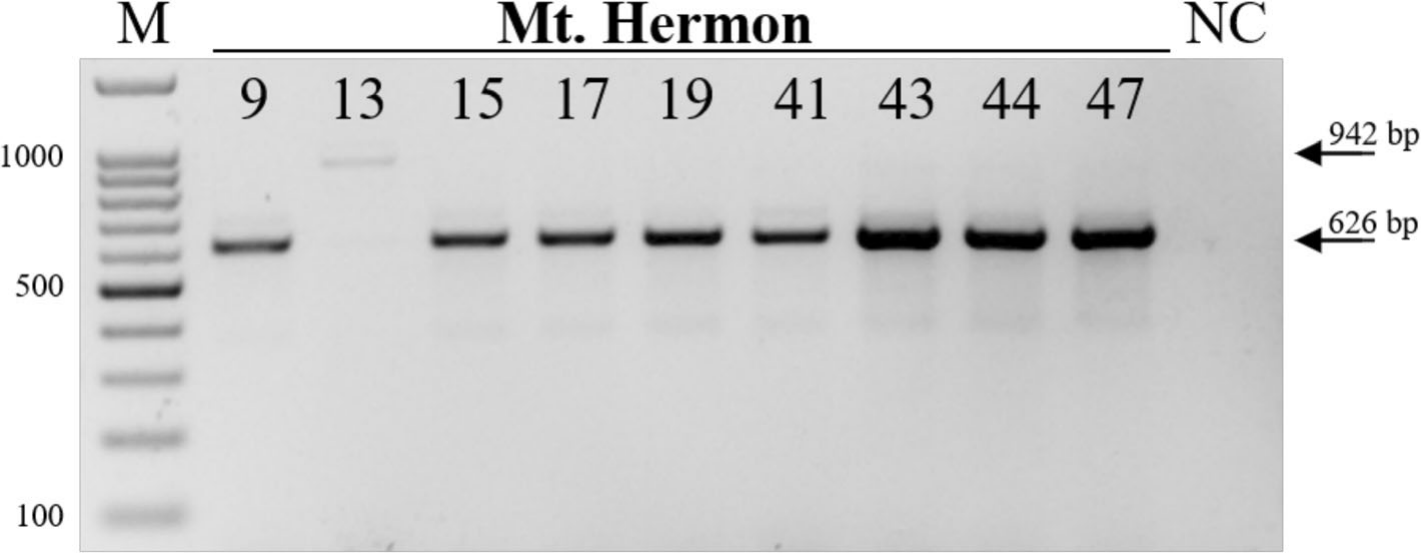
RT-PCR analysis with primers flanking *Mariam* in exon 6 of TRIDC2AG023940 gene. The forward primer (AACCGCAATATGCGGATGTT) was complementary to exon 5 (upstream of *Mariam* insertion), and the reverse primer (TTGTCAATTGTTTGATCAAACAAAGG) was complementary to exon 7 (downstream of *Mariam* insertion). The RT-PCR reaction was performed in the same conditions as the site-specific PCR analysis in Fig. 1 (see Methods). The upper arrow notes a 942 bp band corresponding with *Mariam* insertion (in accession 13), while the lower arrow notes a 626 bp band that does not harbor *Mariam* insertion (in accessions 9, 15–47). M denotes the size marker (the numbers in the left are in bp), while NC denotes a negative control

Amiad, Tabgha and Jaba populations demonstrated over 2-fold differences in expression levels of this gene between some accessions, while the variation within the Mt. Amasa population was less prominent. Mt. Hermon population demonstrated significantly lower expression level than Amiad Population (one-way ANOVA, *F* = 4.3655, *p*-value = 0.0169, Fig. 5). In addition, ~ 2-fold variation was seen within the different accessions in Mt. Hermon population, while the accessions that harbor *Mariam* insertion within the TRIDC2AG023940 gene showed the lowest expressions levels. For example, accession H11 shows reduction in the expression level of TRIDC2AG023940 gene vs. other accessions in Mt. Hermon population. These results might indicate the complex control of the expression of this gene, and do not allow validation or ruling out of the possible effect of this *Mariam* insertion on transcript quantity. However, in this case, the insertion was present in an exon and thus might either interfere with splicing or remain in the mature RNA. To test these possibilities, primers complementary to the predicted cDNA sequence (harbor *Mariam* insertion) were designed for RT-PCR analysis. The expression analysis was performed using cDNA of 9 accessions from the Mt. Hermon population as template. In all examined accessions where no insertion in exon 6 was detected (see Fig. 1), a single clear band corresponding to the expected 626 bp size of the spliced product could be seen (Fig. 6). In the H13 accession where the insertion in exon 6 was present at the DNA level (Fig. 1), there was a single higher band, corresponding to the expected 942 bp size of a spliced product with an insertion in exon 6 (Fig. 6). For validation, this higher band in H13 accession was extracted from the agarose gel, purified and sequenced, and found that this transcript is indeed harboring the full-length *Mariam* insertion. This experiment demonstrates that the insertion in exon 6 altered the mature mRNA sequence produced from the gene in a genome-specific manner.

**Fig. 7:**
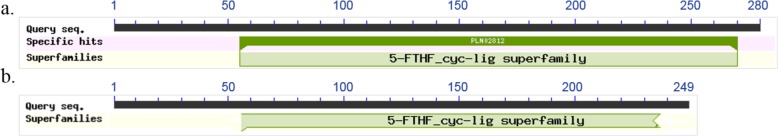
Schematic representation of the conserved protein domains within the coding sequence of TRIDC2AG023940 gene. **a** protein domains of the gene that lacks *Mariam* insertion. **b** protein domains of the gene that harbor *Mariam* insertion in exon 6. This was carried on by a Blastp search in the NCBI database. The numbers on top note amino acid positions. Conserved domains or their parts are marked in g reen. Note that the predicted protein product of the allele harboring *Mariam* insertion is shorter than the predicted product of the regular allele (249 vs. 280 a.a.) and lacks part of the predicted cyclo-ligase domain

According to the coding sequence (CDS) prediction for this gene in the *EnsemblPlants* database, CDS starts in exon 1 and ends in exon 7. Therefore, if the transcript is translated, the new insertion in exon 6 will interfere with translation. ORF analysis for cDNA with the new insertion predicted a stop codon at position 41 out of the 307 nucleotides of the new insertion, so that the protein product produced from the allele with the insertion in exon 6 would possess a shortened and altered C-terminus. A Blastp search of the translated cDNA of the TRIDC2AG023940 gene without the insertion (Fig. 7a) and with the insertion in exon 6 (Fig. 7b) in the NCBI database demonstrated that the insertion interferes with the C-terminal part of the cyclo-ligase domain.

## Discussion

In this study, we present a novel miniature TE-like sequence first discovered in wild emmer wheat. This sequence, initially found in two accessions of Mt. Hermon population of wild emmer wheat and termed *Mariam*, is a low-copy number repetitive sequence. This fragment was found in different wheat species in relatively small numbers, specifically between single insertions in diploid species such as *T. urartu* and in around 12 insertions in wild emmer and bread wheat. The first insertion of *Mariam* discovered, when sequenced, resembled a TE insertion in that it generated a TSD. However, no similar sequence was found in any database of repetitive sequences and TEs. In addition, the small size (307 bp) of *Mariam* indicates that it is non-autonomous and might be classified to miniature inverted-repeat transposable elements (MITEs).

The sequence of *Mariam* does not show any functional similarity to known TEs. It does not possess TIRs that would allow us to assign this element to one of the TIR DNA element super-families. Furthermore, it does not possess any characteristic features of SINEs, such as a Pol-III promotor or a poly-A tail. The only feature that *Mariam* element has in common with a known TE superfamily is the length of the sequence duplications flanking its intact insertions in ~ 50% of cases. Target site duplications (TSDs) that are 9 bp in length are characteristic of *Mutator* TIR DNA elements. It has been proposed that *Mutator* elements might have given rise to MITE derivatives generating 9 bp-long TSDs and that TIRs of some *Mutator* elements might be very short or even missing [6]. Still, all *Mutator*-derived MITEs recently annotated and characterized in grasses possess TIRs (see for examples, [34, 35]). Therefore, while *Mariam* may be a *Mutator*-derived MITE, it is difficult to classify it based solely on its TSD size.

To further investigate the dynamic nature of *Mariam* and to examine its insertional patterns in different wheat species, *Mariam* insertions were retrieved from five publicly available genome drafts of wheat species, and found 33 insertions, some of them were common to wheat species (Additional file 1: Table S1). The bioinformatic analyses showed that *Mariam* is unique to the wheat group and is present in a low copy number in the diploid *T. urartu* (A genome) and *Ae. tauschii* (D genome) species, and therefore most likely was also present in the B progenitor of polyploid wheat. The retrieved *Mariam* insertions of *T. turgidum* ssp. *dicoccoides* were validated on accessions collected from 5 different populations and in 8 accessions of bread wheat, and found high polymorphism levels among accessions, for some cases in a population-specific manner (Table 1).

Some of *Mariam* insertions were found within genic regions, while in most cases *Mariam* was located in the UTR or inside an intron. A2-MH insertion (the first *Mariam* insertion discovered in emmer) was found located within exon 6 of a gene coding for 5-formyltetrahydrofolate cyclo-ligase mitochondrial enzyme. Site-specific PCR analysis showed this insertion was found in only two accessions of Mt. Hermon population of wild emmer wheat, while q-Realtime PCR analysis showed decreased expression of this gene in all Mt. Hermon accessions examined, compared to the other wild emmer wheat populations.

## Conclusions

The discovery of a novel mobile element, a sequence that does not possess any internal characteristics of known TEs, indicates the wheat genome probably possesses other mobile sequences that are difficult to detect, with higher copy number and possibly a prominent functional impact. This study emphasizes the huge contribution of updated genome assemblies to wheat studies while revealing potentially interesting genetic variation in wild emmer wheat populations, as well as in different wheat polyploids. Moreover, although the data collected and analyzed to date does not allow the classification of the newly discovered mobile element, it provides additional insight into the diversity of mobile DNA in wheat genomes.

## Methods

### Genomic and transcriptomic data

We have used the genome drafts of five *Triticum* and *Aegilops* species: (1) *T. urartu*, the donor of A genome (https://www.ncbi.nlm.nih.gov/assembly/GCA_003073215.1) [36]. (2) *Ae. tauschii*, the donor of D genome (https://www.ncbi.nlm.nih.gov/assembly/GCA_000347335.2) [37]. (3) *T. turgidum* ssp. *diccocoides,* wild emmer wheat, genome AB (WEWseq: http://wewseq.wix.com/consortium) [4]. (4) *T. aestivum,* bread wheat, genome ABD. (https://plants.ensembl.org/Triticum_aestivum/Info/Index) [5, 30]. (5) *T. turgidum* ssp. *durum*, durum (pasta) wheat, genome AB [38].

### RNA-seq database

The updated publicly available RNA-seq database of *T. aestivum* and *T. turgidum* ssp. *dicoccoides* found in *Ensemblplants* were used in this study [5, 30]. The library includes cDNA, CDS and ncRNA sequences (https://plants.ensembl.org/Triticum_aestivum/Info/Annotation/, http://plants.ensembl.org/Triticum_dicoccoides/Info/Annotation/).

### Retrieval of *Mariam* insertions

We have used the first *Mariam* sequence identified (A2-MH) as a query in the MITE analysis kit (MAK) software (http://labs.csb.utoronto.ca/yang/MAK/), [39, 40]. MAK is a homology-based software, meaning it uses a consensus sequence as query and BLASTN algorithm with global alignment. We have used an e-value of 1e^− 3^ and an end mismatch tolerance of 20 nucleotides. In addition, flanking sequences (1000 bp from each end) were retrieved to characterize the insertion sites.

### Phylogenetic analysis

Primer6 software version 6.1.6 [41] was used to construct phylogenetic trees clustering the wild emmer and bread wheat accessions according to the insertional polymorphism of *Mariam*, based on ss-PCR. Primer6 software performed hierarchical agglomerative clustering analysis of each matrix with Bray-Curtis similarity and used the similarity profile (SIMPROF) test on each node to assess the statistical significance of the phylogenetic trees. SIMPROF calculates a mean profile by randomizing the value of each variable and re-calculating the profile. The pi statistic was calculated as the deviation of the actual resemblance profile of the resemblance matrix from the mean profile. This was compared with the deviation of further randomly generated profiles to test for significance.

### Statistical analysis

Statistica statsoft was used for the analysis of one-way ANOVA. Relative quantity (relative expression of TRIDC2AG023940 gene by q-realtime-PCR) was used as the dependent variable while Population was used as the categorial variable. Analysis of the specific differences between populations was done using Post hoc and Tukey’s test.

### Plant material and DNA and RNA extraction

A collection of wild emmer wheat (*T. turgidum* ssp. *dicoccoides*) populations from five geographically isolated sites in Israel was used in this study; Mt. Hermon, Amiad, Tabgha, Jaba and Mt. Amasa. The same collection was used in a previous publication [42]. In addition, 8 accessions of *T. aestivum* were used (Additional file 2: Table S2). Seeds were kindly provided by Dr. Sergei Volis (Mt. Hermon, Amiad and Mt. Amasa populations) and by Prof. Eviatar Nevo from the University of Haifa (Tabgha and Jaba populations). Bread wheat accessions were provided by the United States Department of Agriculture (USDA). All wild emmer seeds were provided by Israeli stock centers. Seeds of Tabgha and Jaba Israeli populations are available in the Wild Cereals gene bank (WCGB), Institute of Evolution, University of Haifa. Seeds of Mt. Hermon, Amiad and Mt. Amasa Israeli populations are available in the Lieberman Germplasm Bank, Institute for CerealCrops Improvement, Tel-Aviv University. 10–20 accessions from each population were grown in a greenhouse under common garden conditions. Leaf material was harvested approximately 4 weeks post-germination for DNA extraction using the DNeasy plant mini kit (Qiagen) and for total RNA extraction using TriReagent (Sigma).

### Site-specific PCR

Site-specific PCR primers were designed using Primer3 software (http://bioinfo.ut.ee/primer3-0.4.0/primer3/) to test specific insertion sites. PCR amplifications were prepared with 13.2 μl of Ultra-pure water (HyLabs), 2 μl of 109 Taq DNA polymerase buffer C (EURx), 0.8 μl of 25 mM MgCl_2_ (EURx), 0.8 μl of 2.5 mM dNTP mix, 0.2 μl of Taq DNA polymerase (5 U/μl, EURx), 1 μl of each site-specific primer (50 ng/μl) and 1 μl of genomic DNA (50 ng/μl). The conditions for the PCR reactions were as follows: 94 °C incubation for 3 min, a cycle of (94 °C for 1 min, 57 °C for 1 min, 72 °C for 1 min) repeated 30 times and 72 °C for 3 min. A 10 μl aliquot of the PCR products was tested on a 1.5% agarose gel and visualized with ethidium bromide (Amresco). The expected product sizes were determined by a DNA size standard (100 bp ladder, SMOBIO).

### Single-strand cDNA synthesis

cDNA for gene expression analysis was synthesized using 5xAll-In-One RT MasterMix (abm) in 20 μl reactions. Each reaction contained 4 μl of MasterMix and up to 2 μg of RNA dissolved in 16 μl of Ultra-pure water (HyLabs). The reactions were incubated at 42 °C for 15 min. The purity of each cDNA sample was tested by PCR using site-specific primers complementary to two exons of the *Actin* gene, giving different amplification products for cDNA and genomic DNA. No genomic DNA contamination was detected.

### Gene expression analysis using real-time quantitative RT-PCR

The expression levels of TRIDC2AG023940 were tested by Real-Time quantitative PCR. Primers specific to the cDNA sequence of this gene were designed with Primer Express v2.0 software. One primer was complementary to the exon-exon junction in order to make the reaction less sensitive to possible DNA contamination of RNA samples. The forward primer was designed to span the junction between exons 1 and 2 to ensure that the reaction would not be sensitive to DNA contamination. Note that no pair of genome-specific primers could be optimized for RT-qPCR. Realtime RT-qPCR analysis was then performed on cDNA samples of 19 accessions from the five populations. qPCR experiments were performed using a 7500 Fast Real-Time PCR system and analyzed using the 7500 version 2.0.5 software (Applied Biosystems). Each reaction contained 7.5 μl KAPA SYBR FAST qPCR Master Mix, 0.3 μl ROX Low 509 (Kapa Biosystems), 1 μl forward and 1 μl reverse primers (10 μM), 0.2 μl ddH_2_O and 5 μl template cDNA.

Primers efficiency was tested by Real-Time qPCR with each primer pair using serial dilutions of the template cDNA mix to produce a standard curve. The efficiency was calculated by [(10–1/y) – 1] × 100%, where y is the standard curve slope. Based on the standard curve, a 50-fold dilution of cDNA was used as template in Real-Time qPCR amplifications for gene expression analysis. To validate product specificity, melting curves were produced and demonstrated a single specific product for each primer pair. Real-Time qPCR analysis was performed as described above using the comparative 2^-ΔΔCT^ method, with *Actin* expression levels serving as an endogenous control and one randomly chosen accession as a reference sample. The relative expression levels of the target gene were assessed using the comparative 2^-ΔΔCT^ method, as previously described.

## Supplementary information

**Additional file 1: Table S1.** Intact Mariam insertions retrieved from five wheat species.

**Additional file 2: Table S2.** *T. aestivum* accessions used in this study and their geographic origin.

**Additional file 3: Table S3.** Site-specific primers flanking insertions of *Mariam*.

**Additional file 4: Figure S1.** Sequence logo representing target site preference of *Mariam*. **Figure S2.** Site-specific PCR analyses of *Mariam* insertions.

## Data Availability

All data generated in this study are included in the paper and in the supporting information files.

## Abbreviations

MITE: Miniature inverted-repeat transposable elements
TE: Transposable elements
TIR: Terminal inverted repeat
TSD: Target site duplication
