# Supplementary material for: A novel miniature transposon-like element discovered in the coding sequence of a gene that encodes for 5-formyltetrahydrofolate in wheat
Source: BMC Plant Biol. 2019 Nov 1;19:461. doi: 10.1186/s12870-019-2034-1 (PMC6824096; doi:10.1186/s12870-019-2034-1)
Supplement: Supplementary file 4 — Additional file 4: Figure S1. Sequence logo representing target site preference of Mariam. Figure S2. Site-specific PCR analyses of Mariam insertions. [file 12870_2019_2034_MOESM4_ESM.docx]

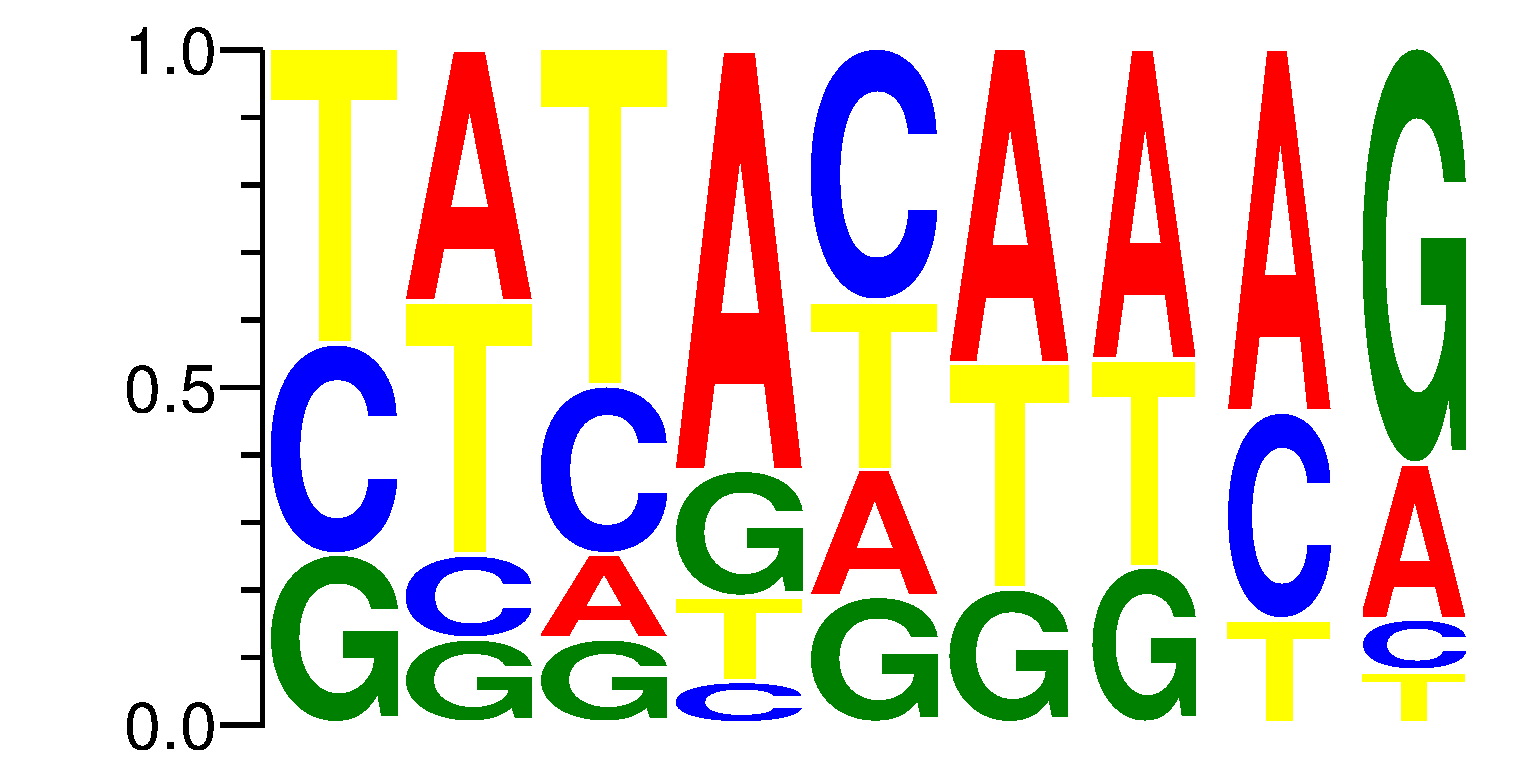


**Fig S1. Sequence logo representing target site preference of *Mariam*.** The logo was created using WebLogo 3.0 package. The letter height notes the probability of each nucleotide at a given position.


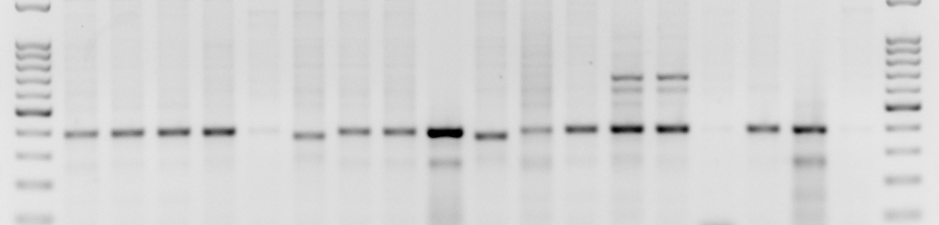

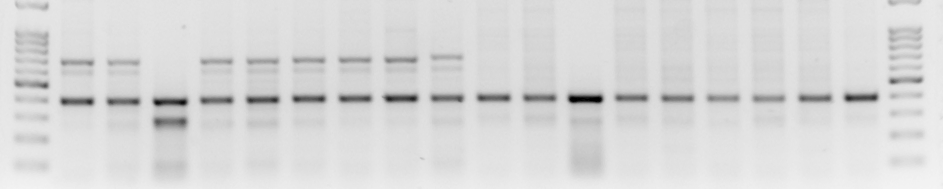

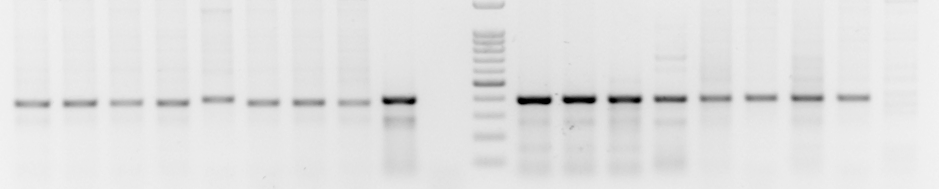


5 10 11 13 19 33 44 46 54 6 7 10 16b 17 18 20 23 28

1 2 3 5 6 7 8 9 10 1 2 3 4 5 7 8 9 10

1 4 8 11 13 16 24 25 89 1 2 3 4 5 6 7 8

M Mt. Hermon Amiad M

M Tabgha Jaba M

Mt. Amasa NC M *T. aestivum*

**a.**


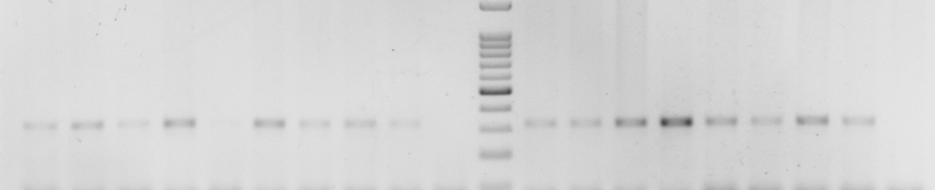

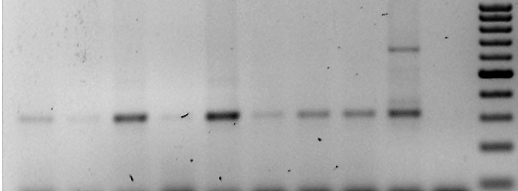

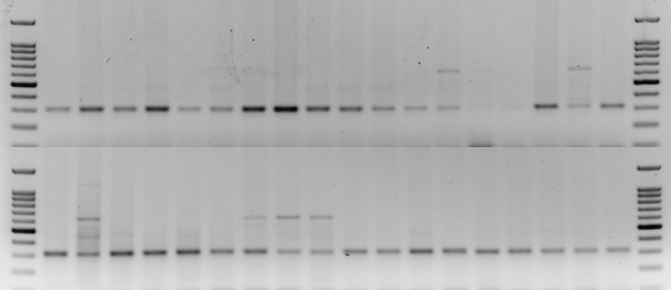

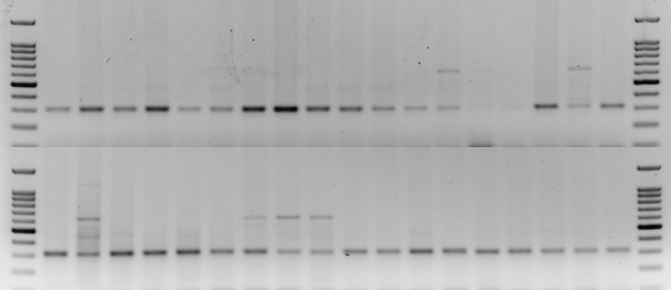


5 10 11 13 19 34 44 46 54 6 7 10 16b 17 18 20 23 28

1 2 3 5 6 7 8 9 10 1 2 3 4 5 7 8 9 10

M Mt. Hermon Amiad M

M Tabgha Jaba M

Mt. Amasa NC M *T. aestivum*

3 4 8 11 13 16 24 25 89 1 2 3 4 5 6 7 8

**b.**


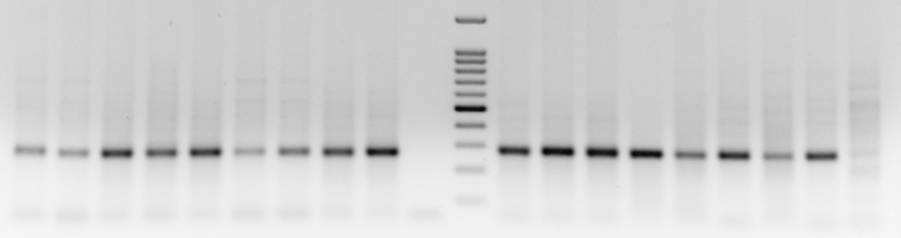

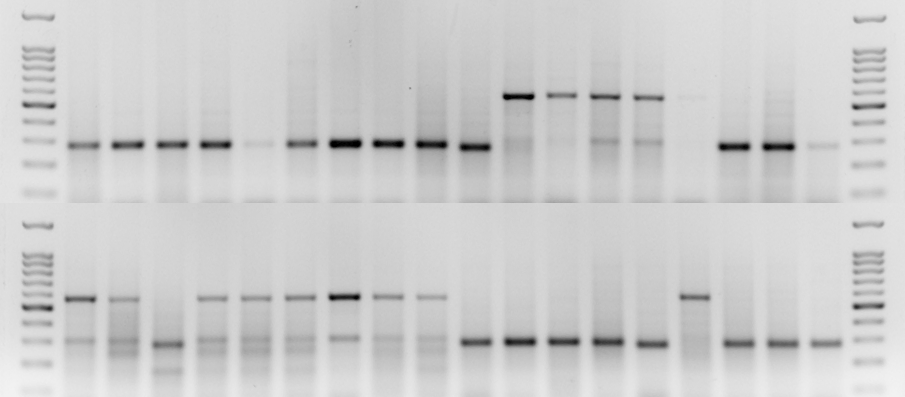

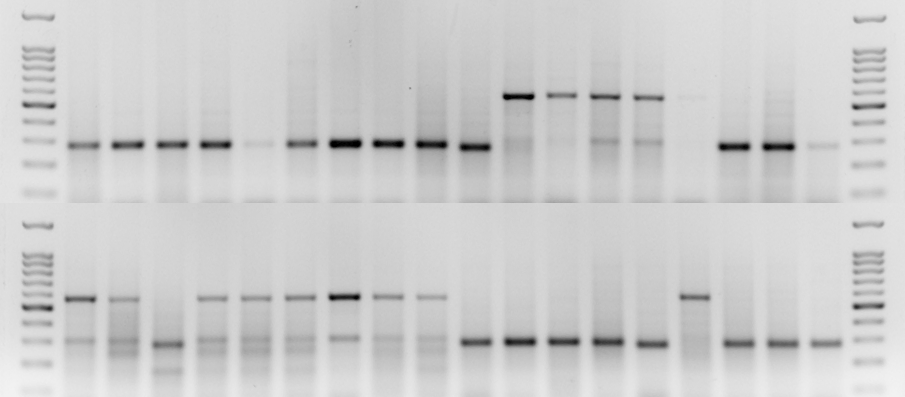


5 10 13 17 19 33 44 46 54 6 7 10 16b 17 18 20 23 28

1 2 3 5 6 7 8 9 10 1 2 3 4 5 7 8 9 10

M Mt. Hermon Amiad M

M Tabgha Jaba M

Mt. Amasa NC M *T. aestivum*

1 3 4 8 11 16 24 25 89 1 2 3 4 5 6 7 8

**d.**


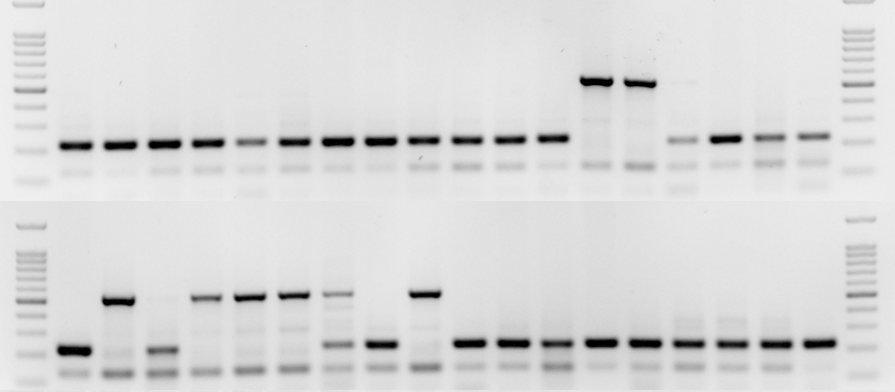

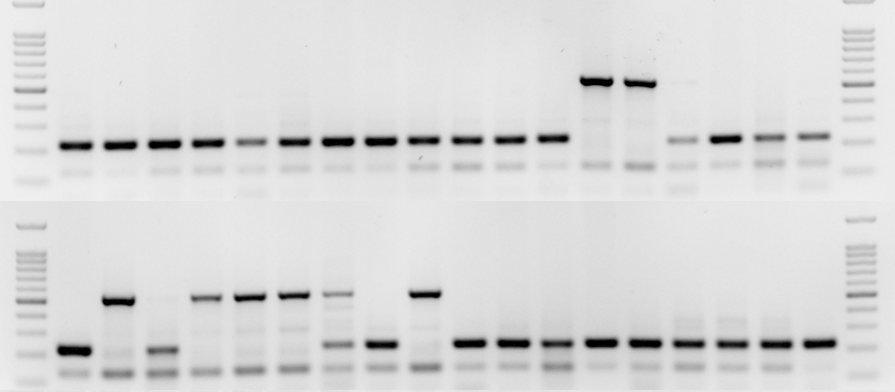

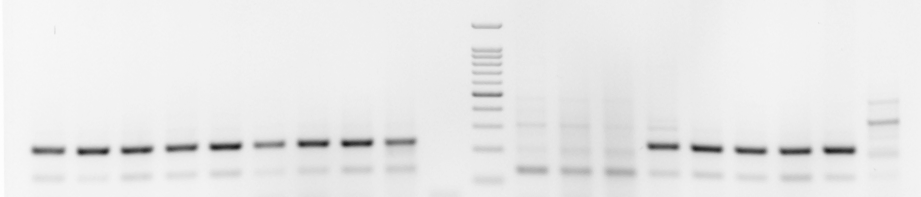


5 10 13 17 19 33 44 46 54 6 7 10 16b 17 18 20 23 28

1 2 3 5 6 7 8 9 10 1 2 3 4 5 7 8 9 10

M Mt. Hermon Amiad M

M Tabgha Jaba M

Mt. Amasa NC M *T. aestivum*

1 3 4 8 11 16 24 25 89 1 2 3 4 5 6 7 8

**c.**


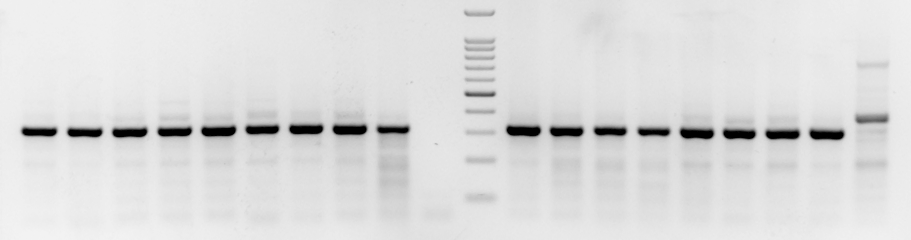

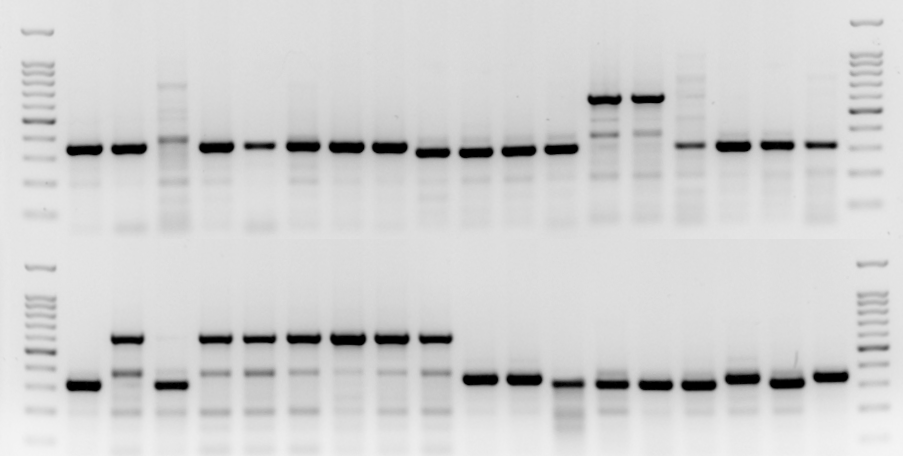

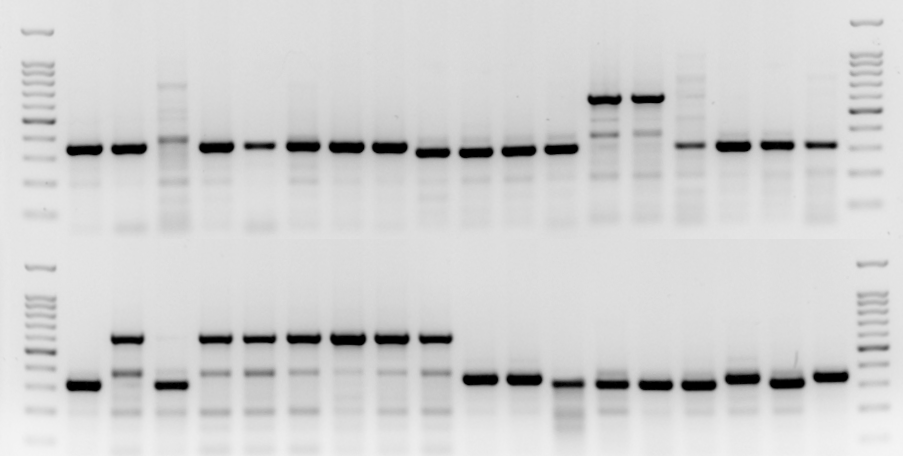


Mt. Amasa NC M *T. aestivum*

5 10 13 17 19 33 44 46 54 6 7 10 16b 17 18 20 23 28

1 2 3 5 6 7 8 9 10 1 2 3 4 5 7 8 9 10

M Mt. Hermon Amiad M

M Tabgha Jaba M

1 3 4 8 11 16 24 25 89 1 2 3 4 5 6 7 8

**e.**


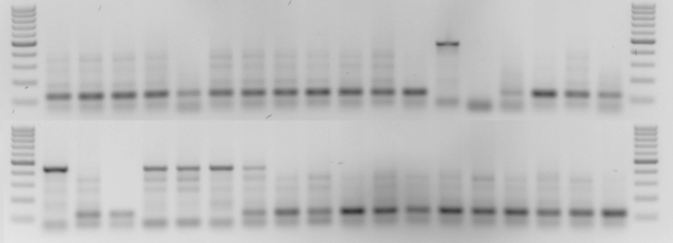

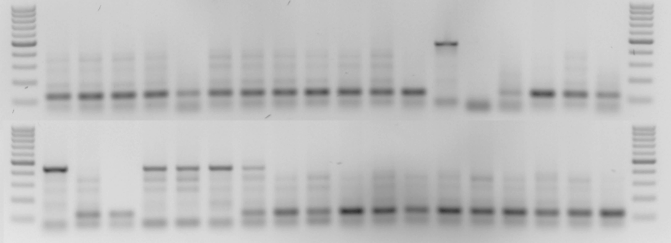

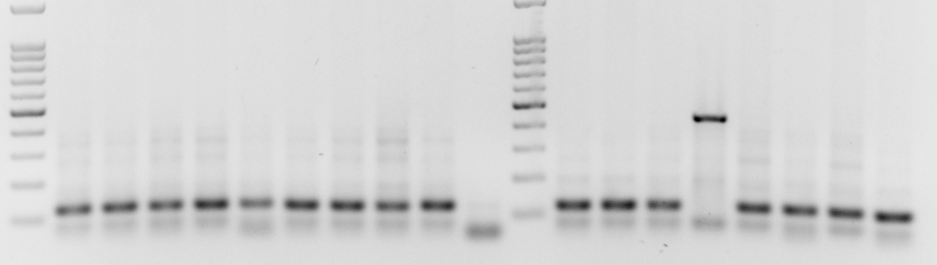


5 10 11 13 19 33 44 46 54 6 7 10 16b * 18 20 23 28

M Mt. Hermon Amiad M

1 2 3 5 6 7 * 9 10 1 2 3 4 5 7 8 9 10

M Mt. Amasa NC M *T. aestivum*

1 4 8 11 13 16 24 25 89 1 2 3 4 5 6 7 8

M Tabgha Jaba M

**f.**


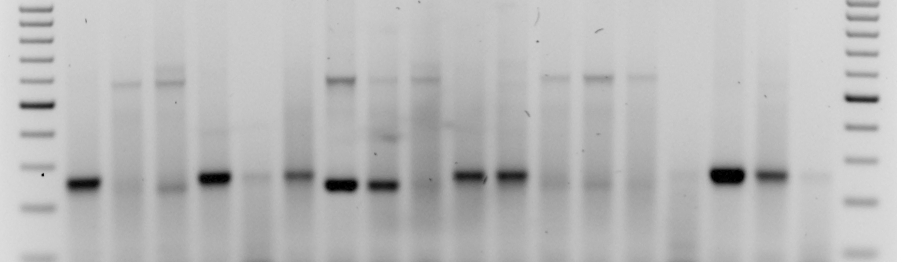


5 10 13 17 19 33 44 46 54 6 7 10 16b 17 18 20 23 28

1 2 3 * 6 7 8 9 10 1 2 3 4 5 7 8 9 10

M Mt. Hermon Amiad M

M Tabgha Jaba M

Mt. Amasa NC M *T. aestivum*

1 3 4 8 11 16 24 25 89 1 2 3 4 5 6 7 8

**g.**

**Fig S2.** **Site-specific PCR analyses of *Mariam* insertions.**
Analyses were performed with 5 populations of wild emmer wheat (top, middle, bottom left) and 8 accessions of bread wheat (bottom right, see additional file 2).
**a, d, e, f** – genome-specific primers; **b, c** – primers complementary to A, B and D subgenomes. Full and empty sites are indicated by arrows. M denotes size marker.
**a** – **A6-2** site: a polymorphic insertion in Amiad and Tabgha populations (some accessions have a 683 bp band indicating full site in addition to the lower band indicating an empty site); all accessions of Mt. Hermon, Jaba, Mt. Amasa and bread wheat have only the lower empty site band (~380-400 bp, its exact length varies between accessions).
**b** – **A7-5** site: a polymorphic insertion in Amiad, Tabgha and Mt. Amasa populations (some accessions have a 639 bp band indicating full site in addition to a 318 bp empty site); all accessions of Mt. Hermon, Jaba and bread wheat have only the lower empty site band.
**c** – **B3-4** site: a polymorphic insertion in Amiad and Tabgha populations (each accession has either 545 bp band indicating full site or 228 bp band indicating empty site); all accessions of Mt. Hermon, Jaba and Mt. Amasa, and five bread wheat accessions (4-8) demonstrate a monomorphic empty site, in other bread wheat accessions no bands of expected size were detected.
**d** – **B7-4** site: a polymorphic insertion in Amiad, Tabgha and Jaba populations (each accession has either 576 bp band indicating full site or a lower band indicating empty site); all accessions of Mt. Hermon and Mt. Amasa and all bread wheat accessions have the lower band indicating an empty site (~270-290 bp, its exact length varies between accessions).
**e** – **B7-6** site: A polymorphic insertion found in some accessions of the Amiad (top right) and Tabgha (middle left) populations indicated by the upper 621 bp band. An empty site (lower 300-320 bp bands) is present in the other Amiad and Tabgha accessions and in all wild emmer from other populations and all bread wheat accessions.
**f** – **A4-1** site: a polymorphic insertion in Amiad and Tabgha populations (each accession has either 437 bp band indicating full site or 119 bp band indicating empty site); the only bread wheat accession demonstrating a full site is TAA01; all accessions of Mt. Hermon, Jaba and Mt. Amasa, and seven bread wheat accessions demonstrate a monomorphic empty site.
**g** – **B1-4** site: a polymorphic insertion in Mt. Hermon, Amiad, Jaba and Mt. Amasa populations as well as bread wheat accessions (each accession has either 575 bp band indicating full site and/or a 261 bp band indicating empty site); all Tabgha accessions have the higher full site band.
